# Association of increased plasma adipocyte fatty acid-binding protein with coronary artery disease in non-elderly men

**DOI:** 10.1186/1475-2840-10-44

**Published:** 2011-05-23

**Authors:** Masayuki Doi, Toru Miyoshi, Satoshi Hirohata, Kazufumi Nakamura, Shinichi Usui, Ko Takeda, Mutsumi Iwamoto, Shozo Kusachi, Kengo Kusano, Hiroshi Ito

**Affiliations:** 1 Department of Cardiology, Kagawa Prefectural Central Hospital, Kagawa, Japan; 2 Department of Cardiovascular Therapeutics, Okayama University Graduate School of Medicine, Dentistry and Pharmaceutical Sciences, Okayama, Japan; 3 Department of Molecular Biology and Biochemistry, Okayama University Graduate School of Medicine, Dentistry and Pharmaceutical Sciences, Okayama, Japan; 4 Department of Cardiovascular Medicine, Okayama University Graduate School of Medicine, Dentistry and Pharmaceutical Sciences, Okayama, Japan; 5 Department of Medical Technology, Okayama University Graduate School of Health Sciences, Okayama, Japan

**Keywords:** adipocyte, fatty acid-binding protein, coronary artery disease, risk factor

## Abstract

**Background:**

Adipocyte fatty acid-binding protein (A-FABP) has been reported to play critical roles in the development of atherosclerosis. We investigated whether an increased in plasma A-FABP level can be independently associated with the presence of coronary artery disease (CAD).

**Methods:**

Two hundred eleven consecutive male patients (mean age: 66 years, range: 33-87 years) were enrolled from inpatients who underwent coronary angiography. Age-matched male subjects (n = 211) having no evidence of CAD served as controls. Plasma A-FABP levels were measured by enzyme-linked immunosorbent assays.

**Results:**

Plasma A-FABP levels in CAD patients were significantly higher than in control subjects (median [IQR], 20.6 [15.7-27.8] ng/mL vs. 15.1 [11.7-19.9] ng/mL, p < 0.01). Multivariate logistic regression analysis revealed that an increased plasma A-FABP level was independently associated with the presence of CAD in all subjects (adjusted odds ratio: 1.76, 95% confidence interval: 1.14 to 2.70, p = 0.01). Furthermore, sub-analysis based on age showed that this association remained significant in subjects aged < 65 years (adjusted odds ratio: 3.06, 95% confidence interval: 1.34 to 6.98, p < 0.01), but not in subjects aged ≥65 years.

**Conclusions:**

Increased plasma A-FABP in non-elderly men had a significant association with the presence of CAD, independent of established CAD risk factors.

## Introduction

Adipocyte fatty acid-binding protein (A-FABP), also known as aP2 or FABP4, is a small intracellular lipid-binding protein [1]. There are nine types of FABPs, showing tissue-specific expression patterns, and A-FABP is abundantly expressed in adipocytes and macrophages [2]. Similar to other FABPs, recent studies showed that A-FABP plays an essential regulatory role in energy metabolism and inflammation [1]. The pathophysiological role of this molecule has been investigated in murine experimental models. A-FABP-deficient mice were protected from the development of insulin resistance in diet-induced obesity[3], type 2 diabetes [4], and atherosclerosis in models of hypercholesterolemia [5]. A-FABP had an effect on atherosclerosis due to not only the dysregulation of systemic metabolism related with adipose tissue, as a result of the activation of macrophages, as it was reported that the expression of A-FABP in macrophages is induced by oxidized low-density lipoprotein (LDL) [6], but also Toll-like receptor activators [7]. It was also reported that an inhibitor of A-FABP markedly reduced atherosclerotic lesions in an ApoE-/- mouse model [5].

Clinically, the involvement of A-FABP in atherosclerosis is supported by a genetic study in human subjects. A carrier of T-87 C polymorphism had lower serum triglyceride levels, demonstrating a reduced cardiovascular risk [8]. Moreover, although A-FABP was originally a cytoplasmic protein, A-FABP levels could be detected in human serum [9]. Higher serum A-FABP has been reported to be useful for the prediction and diagnosis of obesity-related metabolic syndrome and type 2 diabetes mellitus. Previous studies also showed that the serum A-FABP level predicts the development of metabolic syndrome [10] and was associated with carotid intima-media thickness [11], the number of stenotic coronary arteries [12], and coronary plaque volume determined by intravascular ultrasound (IVUS) [13]. These findings demonstrated the role of A-FABP as a potential mediator of atherosclerotic diseases.

Therefore, understanding the clinical significance of circulating levels of A-FABP may be useful for preventing the development of cardiovascular diseases. In this study, we investigated in a large population whether a higher plasma A-FABP level is significantly associated with the presence of CAD after adjustment for established cardiovascular risk factors. We also studied the relationship between A-FABP and CAD according to age.

## Methods

### Study group

This study included 211 consecutive patients (mean age: 66 years, range: 33-87 years) with coronary artery disease (CAD) recruited to undergo coronary angiography, from April 2008 to March 2009, at Kagawa Prefectural Central Hospital, Japan. Patients with CAD had 75% or greater organic stenosis of at least one major coronary artery, confirmed by coronary angiogram, had suffered a myocardial infarction, or had previously undergone percutaneous transluminal coronary angioplasty or coronary artery bypass graft surgery. Patients with hemodialysis, acute coronary syndrome, recent myocardial infarction within 4 weeks, and malignancies were excluded. Control male subjects (n = 211), matched with CAD patients for age (mean age: 66 years, range: 35-85 years), were selected from patients who visited our affiliated hospitals. Controls were characterized by no history of angina or other heart diseases, a normal resting ECG, and normal exercise ECG stress testing. This study protocol complied with the Declaration of Helsinki and was approved by the Ethics Committees of the institute. Informed consent was obtained from all patients before the study entry.

### Clinical and Biochemical assessment

Blood samples were taken after overnight fasting. The plasma was separated and stored at-80°C, and plasma levels of A-FABP (Biovendor Laboratory Medicine, Modrice, Czech Republic) and high sensitivity C-reactive protein (hsCRP; R&D Systems, Minneapolis, MN) were measured by enzyme-linked immunosorbent assay, as previously described[13]. The performance characteristics of these assays were < 7 and < 8% CV intra-assay, and < 5 and < 7% CV inter-assay for A-FABP and hsCRP, respectively.

Risk factors were defined as follows. Diabetes was confirmed according to the criteria of the American Diabetes Association [14], or from a history of diabetes mellitus treatment. Dyslipidemia was defined as one or more of the following criteria: (1) serum triglyceride ≥150 mg/dL, (2) HDL-cholesterol < 40 mg/dL, (3) LDL-cholesterol ≥130 mg/dL, (4) already on lipid-lowering drugs. Hypertension was defined as a resting blood pressure of ≥140/90 mmHg or on regular antihypertensive medications. Smoking was defined as current smokers. eGFR was calculated by the Modification of Diet in Renal Disease (MDRD) equation [15] with coefficients modified for Japanese patients [16]: eGFR (ml/min/1.73 m^2 ^) = 194 × (serum creatinine) ^-1.094 ^× (age) ^- 0.287 ^*Ann Intern Med ***130 **. Renal dysfunction was defined as eGFR < 60 ml/min/1.73 m^2 ^.

### Coronary angiography

Coronary angiography was performed according to standard methods. After intracoronary injection of isosorbide dinitrate, angiograms were obtained in two or more views. The coronary angiogram was scored by two independent investigators and according to three techniques [17]: (1) *Vessel score: *The number of vessels with significant stenosis defined as 50% or greater luminal diameter narrowing. (2) *Stenosis score: *A modified Gensini score, which has been previously reported. Briefly, the most severe stenosis in each of eight segments was graded according to severity, from 1 to 4. The scores in each of the eight segments were added to give a total score out of 32. (3) *Extent score: *According to the proportional length of each vessel segment in the coronary artery tree, segments were graded with different maximum numbers of points, as previously reported. The scores of each vessel were added to give a total score out of 100. Stenosis score and extent score may be regarded to reflect the coronary plaque burden.

### Statistical analysis

Continuous variables are presented as the mean ± SD or median (interquartile range) and differences between two groups were evaluated with an unpaired *t *-test or the Mann-Whitney U test, where appropriate. Categorical variables are presented by frequency counts, and intergroup comparisons were analyzed by the chi-square test. Data that were not normally distributed, determined using the Kolmogorov-Smirrnov test, were logarithmically transformed before linear regression analysis. Differences in plasma A-FABP across the scores of coronary angiography were compared by one-way analysis of variance (ANOVA), followed by the Bonferroni post-hoc test. Associations between CAD and all other parameters were first analyzed by simple logistic regression analysis and then by multivariate analysis. The multivariate adjusted odds ratios (ORs) are presented with 95% confidence intervals (CIs). Statistical significance was defined as p < 0.05. Statistical analysis was performed using SPSS 11.0 for Windows (SPSS Inc., Chicago, IL).

## Results

### Patient characteristics

**Figure 1:**
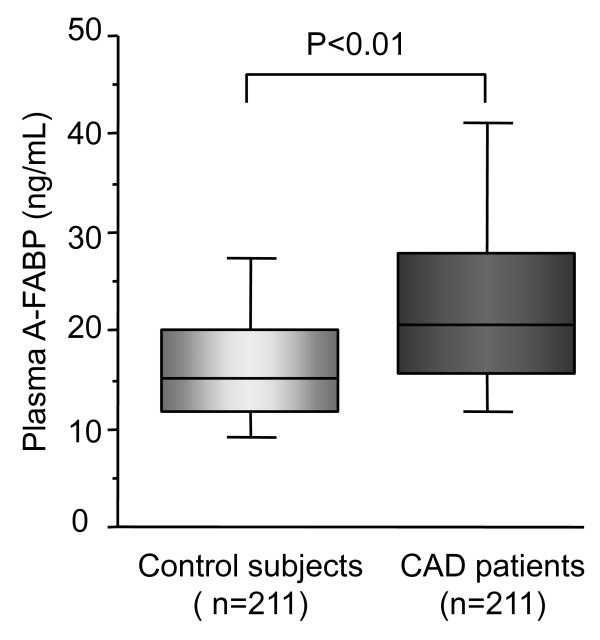
**Box-and whisker plot showing plasma levels of A-FABP in CAD patients and control subjects**. In these plots, lines within boxes represent median values; upper and lower lines in the boxes represent the 25^th ^and 75^th ^percentiles, respectively, and upper and lower lines outside boxes represent the 90^th ^and 10^th ^percentiles, respectively.

Plasma A-FABP levels in CAD patients were significantly higher than in control subjects (median [IQR], 20.6 [15.7-27.8] vs. 15.1 [11.7-19.9], p < 0.001, Figure 1). The clinical characteristics of male CAD patients and control subjects are shown in Table 1. In the fourth quartile (plasma A-FABP level ≥24.5 ng/mL), the number of CAD patients was 2-fold that of control subjects, while in the first quartile, the number of CAD patients were less than that of control subjects. CAD patients had a significantly higher body mass index, fasting blood glucose, LDL cholesterol, and hsCRP, and lower level of HDL cholesterol and eGFR. The presence of hypertension, diabetes mellitus, dyslipidemia, and smoking habit was significantly higher in CAD patients than in control subjects.

**Table 1:** Clinical characteristics of control subjects and CAD patients

	Control Subjects (n = 211)	CAD Patients (n = 211)
Age, years	66 ± 10	66 ± 11
Body mass index, kg/m^2^	23.4 ± 2.9	24.9 ± 3.5*
Smoking, n(%)	29(13)	51(24)*
A-FABP (ng/mL)	15.1 (11.7-19.9)	20.6 (15.7-27.8)*
Diabetes Mellitus, n(%)	39(18)	116(55)*
FBS (mg/dL)	98(92-109)	104(95-126)*
Hypertension, n(%)	65(31)	156(72)*
Dyslipidemia, n(%)	83(39)	162(77)*
LDL-C (mg/dL)	105(85-122)	124(97-134)*
HDL-C (mg/dL)	58 ± 12	43 ± 11*
Triglycerides (mg/dL)	126 (105-151)	129(90-170)
eGFR(ml/min/1.73 m^2^)	75.6 ± 17.6	66.9 ± 18.1*
hsCRP(mg/L)	0.67(0.29-1.68)	1.62(0.60-3.02)*
Medications		
ACEI/ARBs	28(13)	82(39)*
CCBs	44(21)	91(43)*
Diuretics	18(9)	30(14)
β-blockers	23(11)	54(26)*
Statins	55(26)	126(60)*
Thiazolidinediones	15(7)	23(11)

Data are the mean ± SD, median (interquartile range), or frequency counts, as appropriate. A-FABP, adipocyte fatty acid-binding protein; FBS, fasting blood glucose; LDL-C, low-density lipoprotein cholesterol; HDL-C, high-density lipoprotein cholesterol; eGFR, estimated glomerular filtration rate; hsCRP, high sensitivity C-reactive protein; ACEI, angiotensin-converting enzyme inhibitor; ARB, angiotensin II receptor blocker; CCBs, calcium channel blockers.

Among diabetic patients (n = 155), the presence of CAD was higher in those with over the medium level of plasma A-FABP than in those with the lower plasma A-FABP (84% vs. 64%, p < 0.01). Among patients with dyslipidemia (n = 245), the presence of CAD was higher in those with over the medium level of plasma A-FABP than those with lower plasma A-FABP (78% vs. 54%, p < 0.01).

In all subjects, plasma A-FABP was correlated with the body mass index (r = 0.47, p < 0.001), but not with age (r = 0.01, p = 0.86), LDL-cholesterol level (r = 0.07, p = 0.15), or HbA1c (r = 0.09, p = 0.15). Plasma A-FABP in subjects with a history of smoking was significantly higher than that in subjects without a history of smoking (19.6 [15.1-27.2] vs. 17.2 [12.7-24.1], p = 0.03). In each CAD group or control group, medications did not affect the plasma A-FABP level (data not shown).

### Plasma A-FABP and the severity of CAD

**Figure 2:**
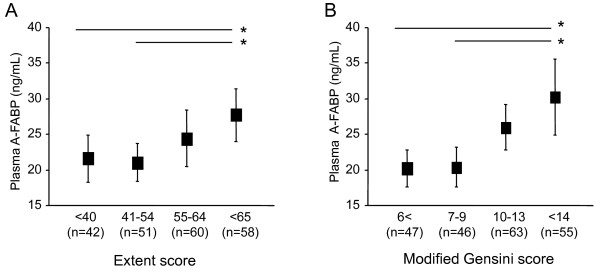
**Association of plasma A-FABP with extent score (A) and modified Gensini score (B) **. Closed boxes: mean; vertical bars: 95%CI. * p < 0.05

The association of plasma A-FABP and three angiographic scores, the vessel score, extent score, and modified Gensini score, were analyzed in CAD patients. The level of plasma A-FABP did not differ among patients with one diseased vessel (20.1 [15.3-28.1]), two diseased vessels (20.9 [15.8-27.4], and three diseased vessels (21.6 [16.7-20.9]). Meanwhile, the extent score and the modified Gensini score were correlated significantly with a stepwise increase in plasma A-FABP (Figure 2).

### Plasma A-FABP and presence of CAD

To assess the relation of each factor with CAD patients, simple logistic regression analysis was performed (Table 2). The body mass index, smoking, plasma A-FABP level, diabetes mellitus, fasting blood glucose, hypertension, dyslipidemia, LDL cholesterol, HDL cholesterol, renal dysfunction, eGFR, and hsCRP were significantly associated with the presence of CAD. The uses of angiotensin-converting enzyme inhibitors/angiotensin II receptor blockers (ACEIs/ARBs), calcium channel blockers (CCBs), β-blockers, and statins were also significantly associated with the presence of CAD statistically; however, the following parameters were dependent on each other; fasting blood glucose and diabetes mellitus, lipid profile and dyslipidemia, and eGFR and renal dysfunction. Therefore, diabetes mellitus, dyslipidemia and renal dysfunction were selected as variates for multiple logistic regression analysis, along with body mass index, smoking, hsCRP, plasma A-FABP, and the use of ACEI/ARBs, CCBs, β-blockers, and statins. Multiple logistic regression analysis, including all the above factors revealed that the increase in plasma A-FABP was independently associated with the presence of CAD (p = 0.01) along with traditional CAD risk factors.

**Table 2:** Logistic regression analysis in whole subjects

	Simple		Multiple	
		
Factors	Crude OR(95%CI)	p	Adjusted OR (95%CI)	p
**Body mass index (per kg/m^2^) **	1.163 (1.090-1.240)	< 0.001	1.024 (0.940-1.117)	0.584
**Smoking (yes) **	2.000 (1.201-3.308)	0.006	2.408 (1.140-2.703)	0.012
**A-FABP (per doubling) **	3.008 (2.158-4.194)	< 0.001	1.755 (1.140-2.703)	0.010
**Diabetes Mellitus (yes) **	5.385 (3.465-9.369)	< 0.001	4.896 (2.799-8.562)	0.001
FBS (per doubling)	11.978 (4.819-29.772)	< 0.001		
**Hypertension (yes) **	6.371 (4.169-9.737)	< 0.001	3.830 (1.755-8.360)	< 0.001
**Dyslipidemia (yes) **	5.099 (3.342-7.779)	< 0.001	1.857 (0.903-3.817)	0.092
LDL-C (per mg/dL)	0.992 (0.986-9.999)	0.024		
HDL-C (per mg/dL)	0.905 (0.887-0.823)	< 0.001		
Triglycerides (per doubling)	1.003 (1.000-1.007)	0.676		
**Rrenal dysfunction (yes) **	2.465 (1.559-3.899)	< 0.001	1.787 (0.946-3.375)	0.074
eGFR (per ml/min/1.73 m^2^)	0.972 (0.961-0.984)	< 0.001		
**hsCRP (per doubling) **	1.379 (1.215-1.564)	< 0.001	1.436 (1.216-1.697)	< 0.001
				

Multiple logistic regression analysis included variates in bold in simple regression analysis and the use of ACEI/ARB, CCBs, β-blockers, and statins. R2 = 0.364 (n = 422)

Furthermore, to evaluate the impact of plasma A-FABP on CAD presence according to age, we analyzed patients aged < 65 years and ≥65 years separately (Table 3). For analysis of subjects aged < 65, age-matched control subjects (n = 92) against CAD subjects aged < 65 years (n = 102) were re-selected from the control group. The mean ages of the control group and CAD group were comparable (57 ± 6 years and 57 ± 6 years, respectively). Simple logistic regression analysis demonstrated that plasma A-FABP, smoking, body mass index, diabetes mellitus, hypertension, dyslipidemia, renal dysfunction, hsCRP, and the use of ACEI/ARBs, CCBs, and statins were significant factors associated with the presence of CAD, Multiple logistic regression analysis including all significant factors in subjects aged < 65 years revealed that the increase in plasma A-FABP was independently associated with the presence of CAD (p = 0.001) along with smoking and diabetes mellitus. Next, for analysis of subjects aged >65, control subjects (n = 93) age-matched against CAD subjects aged >65 years (n = 109) were re-selected from the control group. The mean ages of the control and CAD groups were 75 ± 5 years and 75 ± 6 years, respectively. Simple logistic regression analysis demonstrated that plasma A-FABP, diabetes mellitus, hypertension, dyslipidemia, renal dysfunction, hsCRP, and the uses of ACEI/ARBs, CCBs, β-blockers and statins were significant factors associated with the presence of CAD; however, multiple logistic regression analysis including all significant variates revealed that plasma A-FABP was not an independent factor associated with CAD.

**Table 3:** Significant factors associated with CAD in younger and elder subjects

Subjects aged < 65 years	Adjusted OR (95%CI)	p
A-FABP (per doubling)	3.063 (1.343-6.987)	0.001
Smoking (yes)	2.877 (1.001-8.231)	0.04
Diabetes Mellitus (yes)	6.937 (2.759-17.438)	< 0.001

Subjects aged>65 years	Adjusted OR (95%CI)	p

Diabetes Mellitus (yes)	3.633 (1.602-8.238)	0.002
Hypertension (yes)	4.633 (1.602-8.238)	0.011
hsCRP (per doubling)	1.519 (1.210-1.908)	< 0.001

The model in subjects aged < 65 years included plasma A-FABP, smoking, body mass index, diabetes mellitus, hypertension, dyslipidemia, renal dysfunction, hsCRP, and the use of ACEI/ARBs, CCBs, and statins. The model in subjects aged>65 years included plasma A-FABP, diabetes mellitus, hypertension, dyslipidemia, renal dysfunction, and hsCRP, the use of ACEI/ARBs, CCBs, β-blockers and statins. Only statistical significant factors are presented in Table 3.

## Discussion

In the present study, we demonstrated that higher level of plasma A-FABP in male subjects was independently associated with the presence of CAD after adjustment for established cardiovascular risk factors, such as smoking, diabetes mellitus, dyslipidemia, hypertension, and smoking. Our multivariate logistic analysis demonstrated that the adjusted odds ratio of plasma A-FABP (per doubling) for the presence of CAD was 1.78 (95%CI: 1.14-2.70, p = 0.01). Furthermore, sub-analysis based on age showed that this association remained significant in subjects aged < 65 years, but not in subjects aged ≥65 years.

### A-FABP and CAD

Several lines of evidence have suggested the role of A-FABP in atherogenesis, and it is possible that the high plasma A-FABP levels in patients with CAD in this study were the cause of progressive coronary atherosclerosis. Studies on the molecular function of A-FABP in macrophages have shown that A-FABP deficiency reduced foam cell formation in response to oxidized LDL and increased the cholesterol efflux pathway[18]. A-FABP-deficient mice also showed a significant decrease of vascular atherosclerosis in the absence of differences in serum lipids or insulin sensitivity in hyperlipidemia model mice, and this effect was attributed to the action of A-FABP in macrophages[5]. Thus, A-FABP in macrophages and adipocytes has pathological effects on vessels. In humans, cohort studies suggested that A-FABP plays an important role in insulin resistance and metabolic syndrome[9]. Our study shows that diabetic patients over the medium level of plasma A-FABP had a significantly greater presence of CAD than diabetic patients with lower plasma A-FABP. These results suggest that plasma A-FABP may have a differential impact on CAD from diabetes mellitus. A previous cross-sectional study, however, showed that serum A-FABP was correlated closely with risk factors characterized by abdominal obesity, including insulin resistance, hyperglycemia, increased serum triglyceride, LDL-cholesterol, and decreased HDL-cholesterol[9]. Our findings also found that plasma A-FABP had a weak correlation with hsCRP and eGFR (data not shown). In addition, previous studies showed that medications affect the circulating A-FABP level. Serum A-FABP was reported to be reduced with statin therapy [19], but increased with the treatment of thiazolidinedione[20]. Despite several confounding factors, multiple logistic regression analysis demonstrated that plasma A-FABP was independently associated with the presence of CAD after adjustment for well-known CAD risk factors, hsCRP, and renal dysfunction. Thus, increased plasma A-FABP can be considered a candidate risk factor for CAD, although a large-scale prospective study in the general population is needed.

### Impact of A-FABP on atherosclerosis

A-FABP is expressed in adipocytes and macrophages, and an increase in plasma A-FABP may reflect an increased expression in those cells. Plasma A-FABP concentration has been reported to be correlated with carotid atherosclerotic parameters, such as intima-media thickness[11]and plaque volume of the coronary artery determined with IVUS[13]. In line with previous studies, we demonstrated that plasma A-FABP in CAD patients increased with the severity of angiographic coronary stenosis[12]. Besides the severity of coronary stenosis, previous studies showed that plaque vulnerability is more important for future cardiac events[21]. Extent of atherosclerosis is another marker of an increased risk of coronary events. There is a significant association between coronary artery calcium scores, that reflects the actual presence and severity of atherosclerosis, and cardiovascular events [22]. In our study, A-FABP level was closely associated with modified Gensini score and extent score, that reflect coronary plaque burden. Thus, A-FABP might have potential to be a predictor of cardiovascular outcomes.

Several studies showed that genetic involvement in adipokine regulation was associated with atherosclerosis [23]. Regarding A-FABP, the T-87 C allele in the promoter region of the A-FABP gene has been shown to be associated with lower triglyceride levels, a reduced risk for coronary artery disease and type 2 diabetes [8]. Recent genetic studies identified several candidate genes associated with type 2 diabetes, including KCNQ1 [24], while A-FABP also contributed to the development of diabetes mellitus. In fact, a previous study showed that circulating A-FABP could predict the development of type 2 diabetes [10]. A basic experiment showed that interplay between FABPs and pancreatic islets plays an important role insulin secretion [25]. Thus, even though the direct interaction between A-FABP polymorphism and atherosclerosis has not been fully elucidated, the influence of A-FABP on lipid and glucose metabolism [26]may be one of the important mechanisms of atherosclerosis.

In this study, multivariate analysis using traditional CAD risk factors revealed that the association between elevated plasma A-FABP and the presence of CAD remain significant in patients aged < 65 years, but not in subjects aged ≥65 years. This finding is similar to predictive power of cardiovascular risk factors in relation to aging. Several studies documented that impact of risk factors on mortality decreases with age, partly because of selective survival and the influence of comorbidity on risk factor levels [27-29]. For instance, increased systolic blood pressure or cholesterol in younger subjects may affect cardiovascular mortality events with low-grade elevation or short-term exposure, more than in subjects of advanced age. On the other hand, the effect of each factor for risk control would be reduced in subjects of advanced age. As the progression of coronary artery disease is the manifestation of a cluster of several risk factors, the impact of A-FABP on the development of atherosclerosis would be reduced in subjects of advanced age; thus, the impact of increased plasma A-FABP can be regarded as a coronary risk factor especially in patients aged < 65 years.

### Gender difference in circulating A-FABP

This study focused on men, because male gender is an important risk factor of CAD and gender differences in serum A-FABP have been reported [13]. Previously, we have shown that serum A-FAPB in female patients with CAD was significantly higher than in male patients with CAD, whereas serum A-FABP in men was lower than in women. This contradictory difference in serum A-FABP in women might confuse the analysis of the interaction; therefore, we analyzed only men in this study. This sex difference may be partly the result of the relatively higher fat percentage in women than in men, because adipose tissue is the major contributor of circulating A-FABP. Another explanation is the regulation of A-FABP expression by sex hormones. A study showed that the secretion of adiponectin, which is another major adipokine, was suppressed by testosterone [30]. A similar mechanism of the secretion of A-FABP might be possible. Further studies will be needed to elucidate the clinical implication of increased plasma A-FABP in women in respect of CAD.

## Conclusion

An increase in circulating A-FABP in men was demonstrated to be independently associated with the presence of CAD after adjustment for established cardiovascular risk factors. Further, the plasma A-FABP had more impact on CAD in non-elderly men than in elderly men. Our findings indicated that A-FBAP might be a clinically significant mediator linking obesity and coronary atherosclerosis, and the measurement of circulating A-FABP may be useful to evaluate the risk of CAD.

## Competing interests

The authors declare that they have no competing interests.

No competing interests

## Authors' contributions

MD, TM, KT, MI, conceived the study, and participated in its design and coordination and helped to draft the manuscript.

SU carried out the immunoassays

SH, SK KN, KK, HI were involved in drafting the manuscript or revising it critically

All authors read and approved the final manuscript.
